# Applications of equity frameworks in theory-based health behavior interventions: a scoping review

**DOI:** 10.1186/s12939-025-02438-x

**Published:** 2025-03-20

**Authors:** Katherine S. Gallagher, Kristefer Stojanovski, Kristen Ogarrio, Laura Wright, Melissa Fuster, Caryn N. Bell

**Affiliations:** 1 Department of Health Policy and Management, Celia Scott Weatherhead School of Public Health and Tropical Medicine, Tulane University, New Orleans, USA; 2 Department of Social, Behavioral and Population Sciences, Celia Scott Weatherhead School of Public Health and Tropical Medicine, Tulane University, New Orleans, USA; 3 Department of Epidemiology, Celia Scott Weatherhead School of Public Health and Tropical Medicine, Tulane University, New Orleans, USA; 4 Rudolph Matas Library of Health Sciences, Tulane University, New Orleans, USA

**Keywords:** Health behavior, Theory, Intervention, Equity, Scoping review

## Abstract

**Background:**

Health behavior theories are scientific frameworks used to inform health behavior interventions to address health-related issues, given their use in understanding and modifying behavior change.

**Purpose:**

We aimed to assess how theory-informed health behavior interventions utilize health equity frameworks and methods.

**Methods:**

Using the PRISMA guidelines, we conducted a scoping review of ten often taught health behavior theories found in health behavior textbooks. We identified 656 intervention papers, and after the title and abstract screening and full-text review, we extracted data from 26 studies. We conducted a thematic analysis to examine 1) the prevalence and quality of behavior interventions informed by health equity frameworks and 2) the application of health equity frameworks in the design and implementation of health behavior interventions**.**

**Results:**

Theory-informed health behavior interventions incorporating equity frameworks predominately focused on two strategies. First, by incorporating multilevel frameworks via the socioecological model to influence behavior at multiple levels of risk. The second was utilizing community-based participatory methods to integrate the community’s cultural, social, and lived experiences into the interventions. Creating practices and policies rooted in lived experiences, such as recording meetings, having childcare, and processes for inclusion of feedback served to embed equity into the intervention design and implementation. Studies that were more dedicated to community involvement showed greater community acceptance and improved intervention outcomes.

**Conclusions:**

Our scoping review identified that incorporating equity into health behavior interventions is essential yet not widely practiced and poorly understood regarding how to “bake in equity.” Enhanced training on incorporating equity into theory-informed behavioral interventions could improve health behavior and health education training, research, and practice.

**Supplementary Information:**

The online version contains supplementary material available at 10.1186/s12939-025-02438-x.

## Introduction

In the United States, modifiable health behaviors directly contribute to the five leading causes of death [13]. Literature on health behavior change interventions is plentiful [22]. Highly utilized behavior-change theories, such as the Health Belief Model and the Theory of Planned Behavior, often focus on impacting individual capabilities and motivations [1, 56]. Other models, such as Social Network Theory, attempt to influence social dynamics [17]. While health behavior interventions are necessary, those that acknowledge and address the drivers of inequities (i.e., racism, classism, etc.) could more appropriately influence health behavior. Understanding how to incorporate and address the fundamental social conditions of health equity within health behavior theories and interventions is critical but still not adequately examined.

The social determinants of health refer to the social, economic, political, and legal conditions that shape our lived experiences and health. It is now widely understood that social conditions are fundamental determinants of health inequity [8]; [41, 55]. Health inequities are unjust, unbalanced, and avoidable barriers and obstacles that worsen the health of socially disadvantaged populations. Health equity is a principle of commitment to reduce and eliminate the harmful determinants of health, including the social determinants [9]. Public health agencies like the Centers for Disease Control [14, 40] and Medicare & Medicaid Services [15] have developed health equity frameworks to improve public and population health. The Joint Commission [59] and the National Committee on Quality Assurance [53] accreditation and certification requirements include health equity principles. The ConNECT framework was created in behavioral medicine to practice and foster equity [2]. To practice equity, ConNECT recommends (1) integrating context of people’s lived experiences, (2) fostering a norm of inclusion, (3) ensuring equitable diffusion of innovations, (4) harnessing communication technology, and (5) prioritizing specialized training (ConNECT). Another framework, Remove, Repair, Remediate, Restructure and Provide (R4P), specifically seeks to address health inequities among Black Americans by repairing trust and relationships, restructuring systems to be inclusive, remediating against harm and stigma, and removing barriers that confer disadvantage and thus make things inequitable [31]. Equity frameworks for evaluation have also been developed to ensure community engagement and ownership [63].

Recent reviews have examined how equity is being incorporated into various areas such as governmental public health programs, health systems delivery, and implementation science frameworks and intervention delivery [12, 25, 46]. In the review of government public health programs, health behavior, and education programs were identified as services where equity is applied and could be enhanced [46]. As applied to health behavior and education, equity often took the approach of having community health workers and adapting and culturally tailoring materials to diverse cultural and ethnic groups [46]. Regarding the review of equity in implementation, the use of equity frameworks included studies with explicit incorporation of equity (from design to intervention), others implicit (e.g., use of CBPR), and others implementing the intervention among a priority population (equity group) [25]. While such frameworks exist, the operationalization and application within health behavior theory-informed interventions are unclear.

Health equity frameworks are designed to be operationalized, integrated into, or applied in concert with health behavior change theories *from design to implementation to evaluation* to consider the complex environments in which health behavior interventions are used. The body of research on equity frameworks is growing, and the universe of equity frameworks needs to be better synthesized in their application to health behavior interventions [62]. To define health equity frameworks, we drew from a 2020 Office of Minority Health and Health Equity at the Centers for Disease Control and Prevention (CDC) study. The study highlights tools to inform health equity practice by integrating social, environmental, cultural, and interpersonal factors to guide targeted interventions [40]. We define equity frameworks as those that 1) understand health as a product of complex interactions between people and the environment, 2) critically examine structures that limit or enhance a population’s opportunity to be healthy, and 3) remediate the damage to the health of marginalized populations [40]. In this scoping review, we aim to synthesize how health equity frameworks inform health behavior intervention, design, implementation, and evaluation worldwide.

## Methods

We conducted a scoping review to examine the extent to which health equity frameworks have been applied in health intervention research and to inform practice and future research. We chose a scoping review given the recent increased attention to health equity [52]. We scoped the health behavior intervention and equity framework literature aligned with the Preferred Reporting in Systematic Reviews and Meta-Analyses (PRISMA) criteria (Moher et al., 2009). We focused on ten theories identified through a review of popular health behavior textbooks [18, 23, 27] and those identified from a review of 14-course syllabi as a way to establish “commonly” taught theories (Table 1). The theories were the Health Belief Model, Theory of Reasoned Action, Theory of Planned Behavior, Integrated Behavioral Model, Transtheoretical Model, Social Cognitive Theory, Social Support Theory, Social Network Theory, Theory of Stress and Coping, and Interpersonal Communication Theory.

**Table 1:** Theories taught in social and health behavior courses at schools and programs of public health

SEM	Health Belief Model	Theory of Planned Behavior	Theory of Reasoned Action	Integrated Behavioral Model	Transtheoretical Model	Social Cognitive Theory	Social Network Theory	Theory of Stress and Coping	Interpersonal Communication Theory	Others
**Baylor University, Robbins College of Health and Human** **Course: Theoretical Foundations of Health Behavior and Public Health (PUBH 5315)**										
1	1	1	1	1	1	1		1		Precede/Proceed
**Georgia Southern University (NOT CEPH), Jiann-Ping Hsu College of Public Health** **Social and Behavioral Sciences and Public Health (PUBH 6535)**										
1	1	1	1		1	1	1		1	Precede/Proceed Diffusion of Innovation
**Johns Hopkins University, Bloomberg School of Public Health—Health, Behavior, and Society** **Fundamentals of Health, Behavior, and Society (410.600.01)**										
1										
**New York University (NOT CEPH), School of Global Public Health** **Global Issues in Social and Behavioral Health (GPH-GU 2140–004)**										
1	1	1			1					Precede/Proceed
**Ohio University, Department of Social and Public Health** **Social and Behavioral Sciences in Public Health (HLTH 6720)**										
1	1	1	1		1	1	1	1		Diffusion of Innovation Precede/Proceed
**Purdue University, Department of Public Health** **Theoretical Foundations of Health Behavior (PUBH 602)**										
						1				Diffusion of Innovation Precede/Proceed
**San Jose State University, College of Health and Human Sciences** **Theoretical Foundations of Public Health (PH 271)**										
1	1	1			1	1				
**Syracuse University** **Introduction to Prevention Science (PHP 624)**										
1		1			1	1	1			
**University of Alabama, Department of Health Science** **Theories of Health Behavior (HHE 520)**										
1	1	1	1		1	1	1			Self-Efficacy Theory/Protection Motivation Theory Diffusion of Innovation
**University of California, Davis, Department of Public Health Sciences** **Social and Behavioral Aspects of Public Health (SPH 222)**										
					1		1			
**University of Memphis (NOT CEPH), Division of Social and Behavioral Sciences** **Social and Behavioral Sciences Principles (PUBH 7160)**										
	1	1	1		1	1				
**University of Michigan, School of Public Health** **Psychosocial Factors in Health Related Behavior (HBHE 600)**										
1	1	1	1	1		1		1		Diffusion of Innovation Theory of Fundamental Causes
**University of Minnesota, School of Public Health** **Fundamentals of Social and Behavioral Science (PUBH 6020)**										
1	1	1	1		1	1				Critical Race Theory
**University of North Carolina at Chapel Hill, Gillings School of Global Public Health** **Fundamentals of Social and Behavioral Science (PUBH 6020)**										
1	1	1								

We used the following MESH search terms: behavio*r change (asterisk included to cover the alternative spelling of “behaviour”), intervention, health equity, health disparities, equity frameworks, and social determinants of health. The full MESH search is in the Supplementary Table. We searched in CINAHL (*n* = 112), Global Health (*n* = 53), PubMed (*n* = 174), Scopus (*n *= 229), and Web of Science (*n* = 78) databases and included 10 papers from the review of citations of the Liburd review. We identified 656 studies and removed 330 duplicates, which left 326 studies for title and abstract screening.

The inclusion criteria were as follows: (1) intervention studies; (2) reference one of ten common behavior theories; (3) use an equity framework; (4) measure health or health behaviors; and (5) be published in 2010 or later. The search included records between 2010 and November 2022. We chose 2010 as a period when health equity became a larger policy-driving force. For example, Healthy People 2020 was launched, and the Affordable Care Act was passed in the United States, which both incorporated health disparities as main areas of work [10]. In addition, in 2010, the World Health Organization released its conceptual framework for action on the social determinants of health [66]. The following were the exclusion criteria: (1) not peer-reviewed; (2) not an intervention study (systematic reviews were excluded); (3) no health behavior theory; (4) no equity frameworks; and (5) non-English papers. A systematic review describing common approaches, features, or characteristics of equity frameworks was not available in the literature when we conducted our review, although researchers are presently engaged in this area [62].

**Fig. 1:**
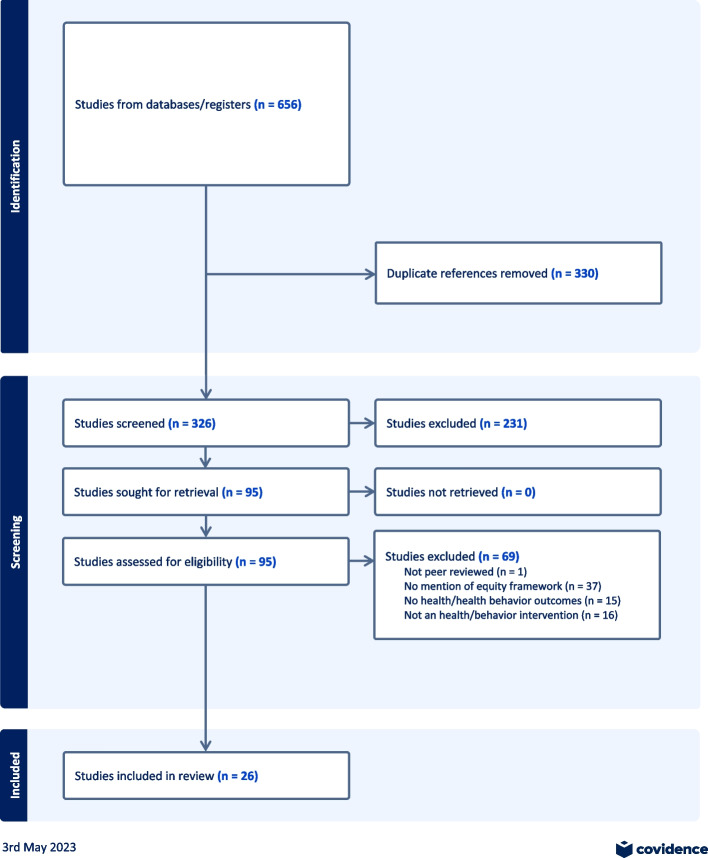
PRISMA flow diagram of scoping review on health equity frameworks in theory-informed health behavior interventions

Two independent reviewers conducted title and abstract screening (KG and KO) in duplicate. Any study that did not meet inclusion criteria after the screening was excluded (*n* = 231), leaving 95 studies for full-text review (reasons for exclusion are not required to be tracked during title and abstract screening). We then conducted a full-text review, and 68 studies were excluded. Reasons for exclusion can be found in Fig. 1. Conflicts were resolved in meetings between the reviewers, where we discussed our voting reasons and came to consensus on whether to include or exclude based on the criteria. Ultimately, 26 papers were included.

KG and KO conducted the data extraction and qualitative content analysis under guidance from the corresponding author. During the full paper review, we extracted (1) author and year of publication, (2) population, (3) geography, (4) study design, and (4) health outcome or health behavior of interest. To capture results and themes, a second table included information extracted on (1) the health behavior theory, (2) equity framework, (3) intervention components, (4) intervention development and design, (5) results of the intervention, and (6) how the intervention integrated theory and utilized equity frameworks.

We conducted a deductive and inductive content analysis [33]. Deductively we used the research protocol and questions as a guide to identify health behavior theories and equity frameworks. Inductively, we explored content across papers in how they defined equity frameworks and their application. KG, KO, and KS developed a set of 25 codes to examine the health behavior theories, equity frameworks, and application of equity frameworks to health behavior interventions. The codes were organized into main themes. During analysis, we used Facilitating Power’s Spectrum of Community Engagement to rank the depth of community engagement utilized in each study from none (score of zero) or limited community involvement (score of one or two) to complete community ownership (score of five) [24].

Risk of bias and quality assessments were completed independently and in duplicate by KG, KO, and KS using different critical appraisal tools depending on study design. For an RCT study design, we used RoB 2 Cochrane risk of bias tool for randomized trials [30], p. 8). ROBINS-I was used to critically appraise non-randomized studies [58], p. 25). See Supplementary File 2 for study characteristics and quality assessments.

## Results

### Overview of papers reviewed

Study characteristics are described in Supplementary Table 2. Most interventions were explicitly focused on racial and ethnic minority groups (*n* = 21). A third of the studies included women only, and four studies involved children as either the sole focus or as part of a family-based intervention. Study designs were pilot or feasibility studies (*n* = 9), randomized controlled trials (*n* = 9), cohort (*n* = 4), and cross-sectional (*n* = 4). There were two dominant categories of health issues or behaviors addressed, diet/lifestyle (*n* = 9) and cancer (*n* = 5).

Most found statistically significant findings when comparing the intervention to the non-intervention groups (*n* = 23). The four studies that did not achieve significance had no or lower levels of community-based methods, such as superficial implementation (e.g., the only translation of study materials to a different language). The 23 significant studies were across various study types and often had higher levels of community engagement (collaborate or defer to).

### Health behavior change theories in the literature

Interventions varied in how deeply activities incorporated theoretical constructs, with most studies (*n* = 20) referencing constructs and how they informed the intervention activities and design [3, 7, 10, 11, 16, 20, 29, 35, 36, 38, 42, 43, 48, 51, 57, 61, 64]. Other studies measured theoretical constructs as outcomes in the intervention (*n* = 17) [3, 7, 10, 11, 16, 20, 29, 35, 36, 38, 42, 43, 48, 51, 57, 61, 64]. Far fewer reviewed studies directly mapped theoretical constructs to specific intervention activities (*n* = 4) [11, 20, 36, 39]. Table 2 outlines the main themes and subthemes of the results. Supplementary Table 3 showcases the theories most predominately identified and other study characteristics.

**Table 2:** Thematic summary of strategies to incorporate equity in health behavior interventions that integrate theory (*n* = 26)

Theme	Subtheme	References
**Role of Theory** *The extent to which theoretical constructs inform intervention activities*	a. Constructs informed activities and design (20)	[3–5, 7, 10, 26, 29, 35–38, 43, 44, 48, 51, 54, 57, 61, 64, 65]
	b. Constructs were measured as outcomes (17)	[3, 7, 10, 11, 16, 20, 29, 35, 36, 38, 42, 43, 48, 51, 57, 61, 64],
	c. Constructs directly applied to intervention activities (4)	[11, 20, 36, 39]
**Consideration of Structural Influences** *How researchers and participants recognize and adapt based on broader structural influences*	a. Multi-level approaches (individual, interpersonal, community, society) (15)	[7, 11, 16, 29, 36–39, 42, 44, 48, 57, 61, 65]
	b. Critical reflections of structural influences (social norms, discrimination oppressive systems, intersectionality, gender roles) (11)	[5, 10, 16, 29, 36–38, 42, 44, 51, 57]
**Community-based Practices** *How community members are organized to drive or support the outcomes of the intervention* *How established principles and practices are used to distribute power, elevate voices, culturally adapt content, and promote co-learning*	a. **Role of Community Members**	
	o Community Advisory Boards (rated on a scale of 1–5 using the Spectrum of Community Engagement) (19)	[3, 4, 7, 10, 11, 26, 29, 35, 36, 38, 39, 42, 44, 48, 51, 57, 60, 64, 65]
	o Community Health Workers (as informants, coordination, delivery, facilitation) (11)	[3, 7, 10, 11, 20, 37, 42, 44, 60, 61]
	o Community Members, Influential leaders (as ambassadors or facilitators) (6)	[7, 37, 38, 48, 60, 64],
	b. **Equity principles**	
	o Established history of partnership (trust, time intensive, prior studies, -OR- lack of trust) (15)	[4, 10, 16, 20, 29, 35–37, 43, 44, 48, 57, 60, 64, 65]
	o Principles for sharing power (transparency, decision making, conflict resolution, co-leading) (8)	[4, 11, 29, 37, 48, 57, 60, 65]
	o Accommodations (scheduling, childcare), compensation for participants (7)	[4, 10, 35, 48, 54, 60, 65]
	c. **Equitable practices**	
	o Materials, activities, and content reflect social norms and values (cultural relevance) (18)	[3, 5, 11, 20, 26, 35–38, 42, 44, 48, 51, 57, 60, 61, 65]
	o Researchers shared characteristics of population (language, ethnicity) (9)	[16, 20, 29, 35, 37, 44, 48, 51, 54]
	o Standardized process for integrating feedback (9)	[3–5, 10, 11, 26, 36, 37, 51]
	o Participant/community involvement in research, methods (literature review, evaluation tools, understanding theory) (6)	[4, 11, 29, 57, 60, 65]
	o Community identified project priorities (5)	[26, 29, 35, 57, 65]
	o Skill-building among community members (ability to address future issues) (2)	[60, 65]

### Use of equity-related frameworks

The most prevalent equity framework applied across the studies was Community-Based Participatory Research (*n* = 21) [3–5, 7, 11, 20, 26, 29, 32, 35–37, 39, 42–44, 48, 57, 60, 64, 65], followed by applications of the Socio-Ecological Model (*n* = 5) [3, 7, 11, 32, 48]. Other frameworks can be found in Supplementary Table 3. The two main ways that equity frameworks were applied were as a: 1) guide for researchers and community members to consider structural influences, and 2) tool for community engagement in intervention development (Table 2).

### Consideration of structural influences

Under consideration of structural influences, interventions attempted to address and mitigate structural influences on health behavior in two main ways: 1) developing multi-level interventions and 2) critically reflecting on how structural factors shape health.

#### Multi-level approaches

Over half of the interventions (*n* = 15), which were all different, used a multi-level approach to consider structural influences (social, economic, cultural, etc.) on health behavior [7, 11, 16, 29, 32, 36–39, 42, 44, 48, 57, 61, 65]. Four of those interventions specifically applied the Socio-Ecological Model (SEM), to influence the social and environmental factors that the individual behaviors are nested within [7, 11, 32, 48]. One faith-based intervention used a multi-level approach to “fit naturally into existing community norms and spaces” by sharing HIV education materials within ministry groups and church services, involving pastors and providing HIV testing during service times [7]. The multi-level intervention produced significant increases in church-based HIV testing. Other interventions used a multi-level approach in stakeholder engagement for intervention delivery. A collaboration with indigenous people in Canada partnered with retailers to increase accessibility of healthy foods, used television and radio spots to expand reach, and held community events to influence factors related to healthy eating and exercise [48]. Another intervention successfully increased cervical cancer screening among low-income Vietnamese women by incorporating multi-level factors such as group-level education sessions, culturally relevant visual aids, and patient navigation services to address individual choices and barriers within the health system [42].

#### Critical reflections on structural influences

A total of 11 interventions reflected on structural factors related to health behavior [5, 10, 16, 29, 36–38, 42, 44, 51, 57]. Critical reflections can help researchers and participants link structural factors (social norms, discrimination, oppressive systems) to health. As one example, researchers used the Chicana Feminist Framework to “link *Promotoras’* experiences to gendered sociocultural scripts for Latina women,” which informed training to balance cultural expectations with boundary setting to benefit the mental health of *promotoras* [5]. In another study, social influencers were central in facilitating conversations with men and women in Benin about family planning – helping to promote learning, diffuse new ideas, and create a welcoming space for discussion of fertility concerns and taboos [38]. In California, discussions of structural influences helped highlight views held by Filipinos regarding colonialism, fatalisms, and resistance, which were used to create an educational video to motivate Filipino parents to support their adolescent’s health [36].

### Community-based practices

Another main theme was the use of equity frameworks via community-based practices, with three subthemes 1) roles of community members, 2) establishment of equity principles, and 3) implementation of equitable practices.

#### Role of community members

Several studies engaged members of the target population and broader community through Community Advisory Boards (CAB) (*n* = 19) [3, 4, 7, 10, 11, 24, 26, 29, 35, 36, 38, 39, 42, 44, 48, 51, 57, 60, 64, 65]. Community experts (typically residents and representatives from community-based organizations) joined structured CABs to ensure interventions were consistent with social and/or cultural norms, preferences, and addressed barriers. All 19 studies that included a CAB were ranked by reviewers as 3 (involved), 4 (collaborate), or 5 (defer to) according to the Spectrum of Community Engagement. In CABs rated as a 3 (involved) (*n* = 6), community members typically aided in providing recommendations for design, implementation, and evaluation, but with ultimate decision-making power lying with the researchers [3, 4, 10, 35, 39, 64]. In CABs rated as a 4 (collaborate) (*n* = 6), community members had more power and influence, often through structured workshop sessions, in determining the content and format of the intervention, including alignment with community priorities and in decisions about expenditure of funds [7, 11, 26, 42, 44, 51]. In CABs rated as a 5 (defer to) (*n* = 7), community members were trained on research methods, were the recipients of funding, and served as leaders of the work, with researchers providing a support role [29, 36, 38, 48, 57, 60, 65].

Community members also contributed to studies in their roles as community health workers (CHWs) (*n* = 11) [3, 7, 10, 11, 20, 32, 37, 42, 44, 60, 61]. CHWs are uniquely positioned for community engagement in health behavior research as trusted members of the community and trained health educators [44]. CHWs provided social and cultural insight during intervention development, in addition to delivering intervention activities and services. In one study, Chinese and Vietnamese CHWs were tasked with recruiting the smoker-family dyads and performing education sessions and helping to build a supportive social network [61]. In another study, church health liaisons (CHLs) were well known in the community and helped coordinate a multi-level HIV testing intervention in alignment with existing church activities and gatherings [7]. In a study to promote cervical cancer screening among Vietnamese women, CHWs facilitated group education sessions, advised in the construction of visual aids, and navigated patients [42].

Finally, community members served as influential community leaders (*n* = 6) [7, 20, 20, 37, 38, 48, 60, 64]. In one study, community members used a mapping exercise to identify influential and connected network actors who could spark discussions and inform community members of the available resources for family planning [38]. In another study, community members were hired and trained to deliver a culturally sensitive diet intervention among indigenous populations in the Canadian Arctic [48].

#### Establishment of equity principles

Equity frameworks provided a foundation for establishing equity principles that reflect the shared values and commitments by researchers and participants. As part of the thematic analysis, a few principles arose, consistent with CBPR principles that promote community-level partnership [40]: 1) trust and established history of partnership, 2) power sharing and transparency, and 3) accommodations and compensation.

##### Trust and established history of partnership

More than half of the interventions had established relationships with the community where the intervention was delivered (*n* = 15) [4, 10, 16, 20, 20, 29, 35–37, 43, 44, 48, 57, 60, 64, 65]. Established partnerships typically extend back between 3 and 5 years, with one dating back 10 years [29]. Such relationships have formed through deep formative research, completion of prior studies, and a devotion to building trust. The review found that such deeply rooted partnerships require significant expenditures of time and resources in early stages. As one study that indicated, “researchers’ ties to the indigenous, patient, and medical communities helped to attain the networks and community support needed for the development and implementation of the intervention” [37]. By contrast, we identified at least two examples of interventions that reported that the lack of adequate time for trust-building hampered success. One school-based intervention to promote physical activity did not describe any prior relationship with the study population, and while the intervention was initially welcomed, it ultimately suffered from lack of continuity and commitment from school leadership and participants [43]. Another intervention to promote endometrial cancer education found that researchers without personal connections to Black women within the community experienced challenges recruiting participants [4].

##### Power sharing and transparency

One third of interventions included principles of power sharing (*n* = 9) [4, 11, 20, 29, 37, 48, 57, 60, 65]. Power-sharing increased transparency in the research process, resolved conflict, and ensured shared decision-making among participants, community members, and researchers. Video recordings of meetings and distribution of meeting minutes improved transparency and ensured groups had access to important information [57]. Explicit commitments to co-learn and co-lead was a recognition that all participants, community members, and researchers bring value to the partnership, should determine its direction, and benefit from its success [29, 48, 60, 65]. One CBPR partnership in Milwaukee created working groups that were co-led by community members and academic partners who developed policies, outreach procedures, and evaluation instruments to improve immunization rates [65]. While many of these partnerships were described as *collaborative*, at least one intervention explicitly described its academic researchers in a *support* role with Latina mothers at the helm [60]. Another group formalized their partnership through a memorandum of understanding, which recognized the relationship as a mutually beneficial exchange of expertise and resources [20]. As a further step of transparency, power sharing, and accountability, one CAB completed an evaluation of the broader group’s adherence to CBPR principles [29]. These examples illustrate the importance of explicit commitments to power sharing by those who have historically held power.

##### Accommodations and compensation

At least seven interventions described accommodations or compensation provided to participants [4, 10, 35, 48, 54, 60, 65]. Accommodations and compensation signal a commitment to equity by recognizing what people give up by choosing to participate in an intervention. Results identified only one example of community members being hired as employers, rather than volunteers [48]. There were other examples of community members being hired into roles as CHWs which require more training. One study designed to reduce obesity among African Americans in California provided accommodations such as free transportation or delivery of healthy food to participant’s homes [54]. Another lifestyle intervention to reduce the risk of diabetes among Filipino Americans hosted educational workshops on the weekend to accommodate working parents [35].

#### Implementation of equitable practices

Equitable practices are the mechanisms through which equity principles are applied. We found the following subthemes: 1) development and use of culturally relevant materials, 2) staffing researchers who share characteristics with the study population, 3) community involvement in intervention, and 4) skill building among community members.

##### Development and use of culturally relevant materials

Two thirds of the studies described the cultural adaptation of materials, activities, and content to reflect social norms and values of the study population (*n* = 18) [3, 5, 11, 20, 26, 32, 35, 35–38, 42, 44, 48, 51, 57, 60, 61, 65]. Some interventions only described how the appearance of the materials was believed to be sufficient adaptations – for example, translating materials into more than one language or featuring individuals of the same racial or ethnic backgrounds as the study population in graphics and videos [3, 26, 32, 42, 61]. Others went beyond the appearance of cultural relevance to build content around commonly held values of the study populations or use cultural references familiar to participants [5, 11, 20, 35–38, 44, 48, 51, 57, 60, 65]. For example, a dance program was designed to improve hypertension management among Native Hawaiians and Pacific Islanders (NHPI). Authors stated that, to be “consistent Native Hawaiian cultural protocol, which necessitates the involvement of informed elders and those with specialized training, six *kumu hula* (hula experts) were interviewed” as part of a process to obtain community-cultural guidance [37]. Another intervention to promote asthma education among Latino populations developed lesson plans based on a culturally appropriate practice of *demostración* (demonstration) circles facilitated by a *promotoras* (CHWs) familiar with community cultures and values that showcases how things are done [60]. Another project to increase stroke awareness incorporated Mexican American values of *familismo* (placing family needs over individual needs) and the social norm of multi-generational households to build an intervention that involved all levels of the family [51].

##### Researchers share characteristics with the study population

Another example of an equity practice is staffing research teams that reflect the study population (*n* = 9) [16, 20, 29, 35, 37, 44, 48, 51, 54]. Employing researchers who are fluent in the study population’s native language or from similar backgrounds was commonly cited as a minimum standard for building rapport. Some had existing ties to the study population, including having grown up in surrounding areas or participated in prior work with the community [29, 35, 37, 48, 51]. Investigators with these shared characteristics were commonly placed in roles that led recruitment, data collection, or facilitation of the intervention.

##### Standardized process for integrating feedback

Nine interventions used a standardized process for integrating feedback from community members [3–5, 10, 11, 26, 36, 37, 51]. One example of a standardized process was the use of Intervention Mapping – systematic process for bridging the gap between theory-based research and the development of appropriate interventions [43]. One group layered several behavioral theories with the socioecological model, CBPR, and intervention mapping in a multi-level intervention to promote cervical cancer screening among Hispanics [11]. In this context, intervention mapping was a helpful tool for systematically engaging community members and effectively tailoring activities to cultural values and norms. Authors shared that “although the CAB looked to the researchers for expertise in behavioral science theory, the health workers and community acted as cultural and linguistic experts, and all members into the acceptability of methods and activities chosen” through the intervention mapping process [11].

##### Participant/community involvement in research methods

Efforts to meaningfully include community members in research processes was another subtheme. Six interventions described various levels of involvement in the research process including processing literature reviews, discussions on behavioral theory, and development of evaluation tools [4, 11, 29, 57, 60, 65]. One study discussed behavioral theory with its CBPR partnership to explore employment and mental health among African American gay men living with HIV/AIDS, which allowed members to understand the process of behavior change and identify where, and how, theory fit into their lived experiences [29]. Other studies (*n* = 4) collaborated on the development of evaluation tools for their intervention, usually through the selection of language used in surveys and to ensure clarity of questions [4, 57, 60, 65]. However, challenges arise with partnerships that are not mutually collaborative and leverage important expertise (whether lived or trained). As one example, the Latina mothers leading an intervention under partnership with a research support team, “missed common pitfalls associated with asking multiple-choice questions such as using the word “except” and double negatives.” [60].

##### Community identified project priorities

Five interventions stated explicitly that the genesis of the project came from community-identified priorities [26, 29, 35, 57, 65]. Many interventions with established partnerships used the years prior to intervention to support the selection of project priorities. One group described the pilot phase as three years “focused on community-in, awareness of the scope of health dipartites, and agreement regarding the research approach, “ultimately focusing their efforts on increasing immunization among children in target zip codes [65]. Another study partnered with a local nurse’s association who had a history of health research and promotion alongside the local Filipino population, who they worked with to identify diabetes risk reduction as a priority [35].

##### Skill-building among community members (ability to address future issues)

Beyond skill-building in research methods, two interventions described a longer-term goal in preparing communities to address broader health disparity concerns [60, 65]. This sentiment reflects a commitment to sustainability and broader transfer of power into the hands of communities. One partnership of Latina mothers described their lessons learned at length, in hopes that their experience could help inform other low-resourced CBPR partnerships [60]. Another group of researchers was thoughtful to embed workshops and more structured trainings during the intervention development, with the goal of technical capacity building, deeper knowledge of health disparities, and advocacy for systems change [65].

## Discussion

Our scoping review showed that equity frameworks in health behavior interventions were incorporated in two ways (1) addressing multi-level factors shaping health behavior and (2) using community-based participatory research methods. However, this was not ubiquitous as our scoping review also found that most studies screened (50%) did not explicitly describe how they incorporated health equity frameworks within health behavior intervention.

While modifiable health behaviors contribute to health, incorporating equity into health behavior interventions is essential. Using HIV as an example, although Black gay, bisexual, and other men who have sex with men are at “highest” risk for HIV, individual-level sexual and drug-using behaviors are not the predominant driving factors, but network (e.g., HIV prevalence within the community) and institutional barriers such as lack of health insurance take precedence [50]. As another example, a conceptual application of Public Health Critical Race Praxis to injury epidemiology showcases how driving (individual-level behavior) can be particularly stress inducing given traffic violations (organizational) and the apparatus of policing structure (structural) shape traffic interactions (interpersonal level) so that such interactions are more violent toward Black Americans [21]. Understanding behavior as an outcome produced within a more extensive system by theories, such as Complex Systems Theory and systems science, may be critical to advance health behavior research, teaching, and practice. Obesity-related research offers a helpful example as it shows how a complex interplay of factors, such as food production, marketing, physical activity, psychology, and physiology, work in tandem to give rise to obesity [19, 28]

A major way that studies in the field attempted to incorporate equity was through community-based participatory research methods (CBPR). CBPR is a research approach that aims to incorporate equity into the research process by ensuring that community voices are integrated into the research process through collaborative decision making. A review of sexual health interventions for racial and ethnic minorities found that studies attributed significant outcomes to collaboration with community members through CBPR [47]. To guide practice, tools such as the Spectrum of Community Engagement may be valuable for transforming public health research and practice by moving away from ignoring (zero on the scale) or tokenizing (two) to deferring to community ownership (five) to strengthen equity and justice ultimately [24]. Within the Spectrum of Community Engagement, community ownership is the goal, which requires creating and fostering democratic participation in research by bridging the divide between community and governance through participatory decision-making. Our review found principles such as power sharing, transparency, participation, trust-building, community leadership, and decision-making are essential for equity within health behavior research. In terms of research, future reviews could incorporate such terms into the search strategies.

The findings from this scoping also have potential implications for health behavior and education training at schools and public health programs. One of the primary disciplines in public health is the social and behavioral health sciences. Foundational coursework in health behavior theory offers a great deal of attention to psychosocial theories. The ten theories chosen for this paper come from health behavior textbooks and course syllabi [18, 23, 27]. One of the competencies of the Council on Education for Public Health Master of Public Health is to “apply awareness of cultural values and practices to the design, implementation, or critique of public health policies or programs.” Introducing equity principles, practices, and frameworks like ConNECT into social and behavior theory coursework can support advancing health behavior education, research, and practice. New, interdisciplinary tools such as design thinking and justice, a human-centered, future-oriented, and problem-solving process, could be additional helpful teaching tools in coursework [6]. Incorporating more systems science theories and research may produce more significant impacts on health equity [45, 49].

As with all studies and reviews, there are limitations. Scoping reviews rely on the underlying evidence as its data; thus, we could not make meta-analytic claims about the effects of CBPR given limited RCTs. However, our findings did show that CBPR has a role in enhancing health behavior interventions. The diversity of study designs was challenging to interpret. RCTs may not be the correct study design for long-term partnerships, community ownership, and equity. Our decision to include feasibility and pilot studies (*n* = 10) means that many papers were ranked as low or critically low quality according to the assessment tools. We included these papers because it was unfair to hold early-stage studies to the standards set forth by the RCT critical appraisal tools and because of the emphasis of our paper on scoping the literature. While we used 10 commonly taught health behavior theories from academic texts, there are countless theories and behavioral areas, such as the 1,275 items found in the Behavioral Change Intervention Ontology developed by the Health Behavior Change Project in areas such as vaccines, travel behavior, vaping, smoking, theatrical communication styles, knowledge acquirement, etc. [34]. Textbook bias is also a limitation as it often includes “typically” taught theories and those chosen as important by the authors. Given that many other health behaviors exist, future reviews should consist of theories such as Diffusion of Innovation, Predisposing, Reinforcing, and Enabling Constructs in Educational Diagnosis and Evaluation and Policy, Regulatory, and Organizational Constructs in Educational and Environmental Development (Precede/Proceed), and the Behavior Change Wheel. Lastly, given that this was a scoping review, the findings are not all-encompassing. Still, they can guide future reviews to examine whether specific health behavior theories may be more appropriate for addressing equity and its linkage to larger public health systems [25, 46].

## Conclusion

Health behavior intervention research, practice, and teaching could benefit from an enhanced direct application of multilevel and community-based methods to improve the application of health equity frameworks. As health inequities grow, equitable health behavior interventions and practices are urgently needed to ensure that participation in healthy behavior is an opportunity for all, with significant ramifications for how equity frameworks are taught to the next generation of practitioners.

## Supplementary Information

Supplementary Material 1.

Supplementary Material 2.

Supplementary Material 3.

## Data Availability

Supplementary and in-text tables contain all the data.
