# Correction: Applications of equity frameworks in theory-based health behavior interventions: a scoping review

**DOI:** 10.1186/s12939-025-02517-z

**Published:** 2025-05-19

**Authors:** Katherine S. Gallagher, Kristefer Stojanovski, Kristen Ogarrio, Laura Wright, Melissa Fuster, Caryn N. Bell

**Affiliations:** 1 Department of Health Policy and Management, Celia Scott Weatherhead School of Public Health and Tropical Medicine, Tulane University, New Orleans, USA; 2 Department of Social, Behavioral and Population Sciences, Celia Scott Weatherhead School of Public Health and Tropical Medicine, Tulane University, New Orleans, USA; 3 Department of Epidemiology, Celia Scott Weatherhead School of Public Health and Tropical Medicine, Tulane University, New Orleans, USA; 4 Rudolph Matas Library of Health Sciences, Tulane University, New Orleans, USA

**Correction: Int J Equity Health 24**,** 79 (2025).**

10.1186/s12939-025-02438-x

Following publication of the original article [1], it was reported that four instances of anonymized text remaining from the peer review process were present in the Methods section.

In the following sentences the bolded text has replaced ‘BLINDED’:

1. Two independent reviewers conducted title and abstract screening (**KG and KO**) in duplicate.
2. **KG and KO** conducted the data extraction and qualitative content analysis under guidance from the corresponding author.
3. **KG**,** KO**,** and KS** developed a set of 25 codes to examine the health behavior theories, equity frameworks, and application of equity frameworks to health behavior interventions.
4. Risk of bias and quality assessments were completed independently and in duplicate by **KG**,** KO**,** and KS** using different critical appraisal tools depending on study design.

The original article [1] has been corrected.
