# Supplementary material for: Applications of equity frameworks in theory-based health behavior interventions: a scoping review
Source: Int J Equity Health. 2025 Mar 20;24:79. doi: 10.1186/s12939-025-02438-x (PMC11924764; doi:10.1186/s12939-025-02438-x)
Supplement: Supplementary file 1 — Supplementary Material 1. [file 12939_2025_2438_MOESM1_ESM.docx]

**Supplementary Table 1: Full search terms and logic per database**

**PubMed**

| Search # | Subject | Search Strategy | # of Results |
| --- | --- | --- | --- |
| 1 | Health equity framework | (((((((((((((((((((((((((((("Health Equity"[Mesh]) OR (health equity[Title/Abstract])) OR (health equity framework[Title/Abstract])) OR (health equity frameworks[Title/Abstract])) OR (health equity strategy[Title/Abstract])) OR (health equity strategies[Title/Abstract])) OR ("Community-Based Participatory Research"[Mesh])) OR (community based participatory research[Title/Abstract])) OR (consumer driven community based research[Title/Abstract])) OR (CBRP[Title/Abstract])) OR ("Health Inequities"[Mesh])) OR (health inequity[Title/Abstract])) OR (health inequities[Title/Abstract]))) OR ("Health Status Disparities"[Mesh])) OR (health disparity[Title/Abstract])) OR (health disparities[Title/Abstract])) OR (health status disparity[Title/Abstract])) OR (health status disparities[Title/Abstract])) OR (social determinant of health[Title/Abstract])) OR (social determinants of health[Title/Abstract])) OR (health social determinant[Title/Abstract])) OR (health social determinants[Title/Abstract])) OR (structural determinant of health[Title/Abstract])) OR (structural determinants of health[Title/Abstract])) OR (health structural determinant[Title/Abstract])) OR (health structural determinants[Title/Abstract])) OR (R4P[Title/Abstract])) OR (ConNECT framework[Title/Abstract]) | 57833 |
| 2 | Theories | ((((((((((("Health Belief Model"[Mesh]) OR ("Transtheoretical Model"[Mesh])) OR (Health Belief Model[Title/Abstract])) OR (Theory of Reasoned Action[Title/Abstract])) OR (Theory of Planned Behavior[Title/Abstract])) OR (Integrated Behavioral Model[Title/Abstract])) OR (Transtheoretical Model[Title/Abstract])) OR (Social Cognitive Theory[Title/Abstract])) OR (Social Support Theory[Title/Abstract])) OR (Social Network Theory[Title/Abstract])) OR (Theory of Stress[Title/Abstract] AND Coping[Title/Abstract])) OR (Interpersonal Communication Theory[Title/Abstract]) | 10749 |
| 3 | Interventions | (((((((((((((((((((((((("Public Health Practice"[Mesh]) OR (public health practice[Title/Abstract])) OR (public health practices[Title/Abstract])) OR ("Health Behavior"[Mesh])) OR (health behavior[Title/Abstract])) OR (health behaviour[Title/Abstract])) OR (behavior change[Title/Abstract])) OR (behavior changes[Title/Abstract])) OR (behaviour change[Title/Abstract])) OR (behaviour changes[Title/Abstract])) OR (behavior change theory[Title/Abstract])) OR (behavior change theories[Title/Abstract])) OR (behaviour change theory[Title/Abstract])) OR (behaviour change theories[Title/Abstract])) OR (intervention[Title/Abstract])) OR (interventions[Title/Abstract])) OR (program[Title/Abstract])) OR (programs[Title/Abstract])) OR (programming[Title/Abstract])) OR (campaign[Title/Abstract])) OR (campaigns[Title/Abstract])) OR (project[Title/Abstract])) OR (projects[Title/Abstract])) OR (workshop[Title/Abstract])) OR (workshops[Title/Abstract]) | 3066607 |
| 4 | FULL | #1 AND #2 AND #3 | 185 |
| 5 | FULL w/time limits | #1 AND #2 AND #3 | 174 |
| 6 | FULL w/time limits | (((((((((((((((((((((((((((((("Health Equity"[Mesh]) OR (health equity[Title/Abstract])) OR (health equity framework[Title/Abstract])) OR (health equity frameworks[Title/Abstract])) OR (health equity strategy[Title/Abstract])) OR (health equity strategies[Title/Abstract])) OR ("Community-Based Participatory Research"[Mesh])) OR (community based participatory research[Title/Abstract])) OR (consumer driven community based research[Title/Abstract])) OR (CBRP[Title/Abstract])) OR ("Health Inequities"[Mesh])) OR (health inequity[Title/Abstract])) OR (health inequities[Title/Abstract]))) OR ("Health Status Disparities"[Mesh])) OR (health disparity[Title/Abstract])) OR (health disparities[Title/Abstract])) OR (health status disparity[Title/Abstract])) OR (health status disparities[Title/Abstract])) OR (social determinant of health[Title/Abstract])) OR (social determinants of health[Title/Abstract])) OR (health social determinant[Title/Abstract])) OR (health social determinants[Title/Abstract])) OR (structural determinant of health[Title/Abstract])) OR (structural determinants of health[Title/Abstract])) OR (health structural determinant[Title/Abstract])) OR (health structural determinants[Title/Abstract])) OR (R4P[Title/Abstract])) OR (ConNECT framework[Title/Abstract])) AND (((((((((((("Health Belief Model"[Mesh]) OR ("Transtheoretical Model"[Mesh])) OR (Health Belief Model[Title/Abstract])) OR (Theory of Reasoned Action[Title/Abstract])) OR (Theory of Planned Behavior[Title/Abstract])) OR (Integrated Behavioral Model[Title/Abstract])) OR (Transtheoretical Model[Title/Abstract])) OR (Social Cognitive Theory[Title/Abstract])) OR (Social Support Theory[Title/Abstract])) OR (Social Network Theory[Title/Abstract])) OR (Theory of Stress[Title/Abstract] AND Coping[Title/Abstract])) OR (Interpersonal Communication Theory[Title/Abstract]))) AND ((((((((((((((((((((((((("Public Health Practice"[Mesh]) OR (public health practice[Title/Abstract])) OR (public health practices[Title/Abstract])) OR ("Health Behavior"[Mesh])) OR (health behavior[Title/Abstract])) OR (health behaviour[Title/Abstract])) OR (behavior change[Title/Abstract])) OR (behavior changes[Title/Abstract])) OR (behaviour change[Title/Abstract])) OR (behaviour changes[Title/Abstract])) OR (behavior change theory[Title/Abstract])) OR (behavior change theories[Title/Abstract])) OR (behaviour change theory[Title/Abstract])) OR (behaviour change theories[Title/Abstract])) OR (intervention[Title/Abstract])) OR (interventions[Title/Abstract])) OR (program[Title/Abstract])) OR (programs[Title/Abstract])) OR (programming[Title/Abstract])) OR (campaign[Title/Abstract])) OR (campaigns[Title/Abstract])) OR (project[Title/Abstract])) OR (projects[Title/Abstract])) OR (workshop[Title/Abstract])) OR (workshops[Title/Abstract])) | 174 |

**Web of Science**

| Search # | Subject | Search Strategy | # of Results |
| --- | --- | --- | --- |
| 1 | Health equity framework | “health equity” OR “health equity framework*” OR “health equity strateg*” OR “community based participatory research” OR “consumer driven community based research” OR “CBRP” OR “health inequit*” OR “health disparit*” OR “health status disparit*” OR “social determinant* of health” OR “health social determinant*” OR “structural determinant* of health” OR “health social determinant*” OR “R4P” OR “ConNECT framework” (Title) or “health equity” OR “health equity framework*” OR “health equity strateg*” OR “community based participatory research” OR “consumer driven community based research” OR “CBRP” OR “health inequit*” OR “health disparit*” OR “health status disparit*” OR “social determinant* of health” OR “health social determinant*” OR “structural determinant* of health” OR “health social determinant*” OR “R4P” OR “ConNECT framework” (Abstract) | 30661 |
| 2 | Theories | “health belief model” OR “theory of reasoned action” OR “theory of planned behavior” OR “integrated behavioral model” OR “transtheoretical model” OR “social cognitive theory” OR “social support theory” OR “social network theory” OR “theory of stress and coping” OR “interpersonal communication theory” (Title) or “health belief model” OR “theory of reasoned action” OR “theory of planned behavior” OR “integrated behavioral model” OR “transtheoretical model” OR “social cognitive theory” OR “social support theory” OR “social network theory” OR “theory of stress and coping” OR “interpersonal communication theory” (Abstract) | 16375 |
| 3 | Interventions | “public health practice*” OR “health behavio*r” OR “behavio*r change*” OR “behavio*r change theor*” OR “intervention*” OR “program*” OR “campaign*” OR “project*” OR “workshop*” (Title) or “public health practice*” OR “health behavio*r” OR “behavio*r change*” OR “behavio*r change theor*” OR “intervention*” OR “program*” OR “campaign*” OR “project*” OR “workshop*” (Abstract) | 3786661 |
| 4 | FULL | #1 AND #2 AND #3 | 86 |
| 5 | FULL w/limits | #1 AND #2 AND #3 | 78 |

**CINAHL**

| Search # | Subject | Search Strategy | # of Results |
| --- | --- | --- | --- |
| 1 | Health equity framework | ( (MH "Action Research") OR (MH "Health Status Disparities") OR (MH "Social Determinants of Health") OR (MH "Health Inequities") ) OR TI ( “health equity” OR “health equity framework*” OR “health equity strateg*” OR “community based participatory research” OR “consumer driver community based research” OR “CBRP” OR “health inequity” OR “health inequities” OR “health disparit*” OR “health status disparit*” OR “social determinant* of health” OR “health social determinant*” OR “structural determinant* of health” OR “health structural determinants” OR “R4P” OR “ConNECT framework” ) OR AB ( “health equity” OR “health equity framework*” OR “health equity strateg*” OR “community based participatory research” OR “consumer driver community based research” OR “CBRP” OR “health inequity” OR “health inequities” OR “health disparit*” OR “health status disparit*” OR “social determinant* of health” OR “health social determinant*” OR “structural determinant* of health” OR “health structural determinants” OR “R4P” OR “ConNECT framework” ) | 40787 |
| 2 | Theories | ( (MH "Health Belief Model") OR (MH "Ajzen-Fishbein Theory of Reasoned Action") OR (MH "Ajzen's Theory of Planned Behavior") OR (MH "Transtheoretical Stages of Change Model") OR (MH "Bandura's Social Cognitive Theory") OR (MH "Lazarus Theory of Stress and Coping") ) OR TI ( “health belief model” OR “theory of reasoned action” OR “theory of planned behavior” OR “integrated behavioral model” OR “transtheoretical model” OR “social cognitive theory” OR “social support theory” OR “social network theory” OR “theory of stress and coping” OR “interpersonal communication theory” ) OR AB ( “health belief model” OR “theory of reasoned action” OR “theory of planned behavior” OR “integrated behavioral model” OR “transtheoretical model” OR “social cognitive theory” OR “social support theory” OR “social network theory” OR “theory of stress and coping” OR “interpersonal communication theory” ) | 12778 |
| 3 | Interventions | ( (MH "Health Behavior") OR (MH "Behavioral Changes") ) OR TI ( “public health practice*” OR “health behavio*r” OR “behavio*r change*” OR “behavio*r change theor*” OR “intervention*” OR “program*” OR “campaign*” OR “project*” OR “workshop*” ) OR AB ( “public health practice*” OR “health behavio*r” OR “behavio*r change*” OR “behavio*r change theor*” OR “intervention*” OR “program*” OR “campaign*” OR “project*” OR “workshop*” ) | 1036734 |
| 4 | FULL | #1 AND #2 AND #3 | 137 |
| 5 | FULL w/time limits | #1 AND #2 AND #3 | 112 |

**Global Health**

| Search # | Subject | Search Strategy | # of Results |
| --- | --- | --- | --- |
| 1 | Health equity frameworks | health inequalities/ or (health equity or health equity framework* or health equity strateg* or community based participatory research or consumer driven community based research or CBRP or health inequit* or health disparit* or health status disparit* or social determinant* of health or health social determinant* or structural determinant* of health or health social determinant* or R4P or ConNECT framework).ti,ab. | 19634 |
| 2 | Theories | (health belief model or theory of reasoned action or theory of planned behavior or integrated behavioral model or transtheoretical model or social cognitive theory or social support theory or social network theory or (theory of stress and coping) or interpersonal communication theory).ti,ab. | 4031 |
| 3 | Interventions | (public health practice* or health behavio*r or behavio*r change* or behavio*r change theor* or intervention* or program* or campaign* or project* or workshop*).ti,ab. | 528381 |
| 4 | FULL | #1 AND #2 AND #3 | 58 |
| 5 | FULL w/limits | #1 AND #2 AND #3 | 53 |

**Scopus**

| Search # | Subject | Search Strategy | # of Results |
| --- | --- | --- | --- |
| 1 | Health equity framework | TITLE-ABS-KEY ( *"health equity"*  OR  *"health equity framework*"*  OR  *"health equity strateg*"*  OR  *"community based participatory research"*  OR  *"consumer driven community based research"*  OR  *"CBRP"*  OR  *"health inequit*"*  OR  *"health disparit*"*  OR  *"health status disparit*"*  OR  *"social determinant* of health"*  OR  *"health social determinant*"*  OR  *"structural determinant* of health"*  OR  *"health social determinant*"*  OR  *"R4P"*  OR  *"ConNECT framework"* ) | 81623 |
| 2 | Theories | TITLE-ABS-KEY ( "health belief model"  OR  "theory of reasoned action"  OR  "theory of planned behavior"  OR  "integrated behavioral model"  OR  "transtheoretical model"  OR  "social cognitive theory"  OR  "social support theory"  OR  "social network theory"  OR  "theory of stress and coping"  OR  "interpersonal communication theory" ) | 30429 |
| 3 | Interventions | TITLE-ABS-KEY ( *"public health practice*"*  OR  *"health behavio*r"*  OR  *"behavio*r change*"*  OR  *"behavio*r change theor*"*  OR  *"intervention*"*  OR  *"program*"*  OR  *"campaign*"*  OR  *"project*"*  OR  *"workshop*"* ) | 7793809 |
| 4 | FULL | #1 AND #2 AND #3 | 243 |
| 5 | FULL w/limits | #1 AND #2 AND #3 | 229 |
