# Supplementary material for: Applications of equity frameworks in theory-based health behavior interventions: a scoping review
Source: Int J Equity Health. 2025 Mar 20;24:79. doi: 10.1186/s12939-025-02438-x (PMC11924764; doi:10.1186/s12939-025-02438-x)
Supplement: Supplementary file 2 — Supplementary Material 2. [file 12939_2025_2438_MOESM2_ESM.docx]

**Supplementary table 2: Study characteristics of included studies (n=26)**

| Title | Author, Year, | Population | Geography | Study Design | Health Outcome or Behavior of Interest |
| --- | --- | --- | --- | --- | --- |
| A pilot test of a church-based intervention to promote multiple cancer-screening behaviors among Latinas | Allen (2014) | Latinas ages 18+ | Boston, Massachusetts, United States | Pre-post, 1-arm | Cancer screening |
| "We are a powerful movement”: evaluation of an endometrial cancer education program for black women | Alson (2021) | Black women, EC survivors | United States | Pre-post | Cancer education |
| "No Queremos Quedar Mal": A Qualitative Analysis of a Boundary Setting Training Among Latina Community Health Workers | Alvarez-Hernandez (2021) | Latina, immigrant community health workers | Southeastern United States | Observational, Cohort, Qualitative | Boundary setting, mental health |
| Feasibility and Outcomes of an HIV Testing Intervention in African American Churches | Berkley-Patton (2019) | African American Adults | Kansas City, United States | Cluster Randomized Controlled Trial | HIV testing |
| The development of an innovative, theory-driven, psychoeducational HIV/STI prevention intervention for heterosexually active Black adolescents with mental illnesses | Brawner (2019) | Black adolescents with mental illness | Philadelphia, PA, United States | Pilot Randomized Controlled Trial | HIV/STI Prevention |
| Group Versus Individual Culturally Tailored and Theory-Based Education to Promote Cervical Cancer Screening Among the Underserved Hispanics: A Cluster Randomized Trial | Calderón-Mora (2020) | Hispanic women ages 21-65, uninsured | Texas, United States | Cluster Randomized Controlled Trial | Cervical cancer screening |
| Group Dance and Motivational Coaching for Walking: A Physical Activity Program for South Asian Indian Immigrant Women Residing in the United States | Daniel (2021) | South Asian Indian Immigrant Women | N/A | Longitudinal, 2-arm | Physical activity |
| Educating Hispanic Women about Cervical Cancer Prevention: Feasibility of a Promotora-Led Charla Intervention in a Farmworker Community | Fleming (2018) | Hispanic women | United States | Pre-post, 1-arm | Cervical cancer prevention |
| Dash of Faith: A Faith-Based Participatory Research Pilot Study | Harmon (2014) | African Americans | United States | Quasi-experimental | Diet, Weight Loss |
| A pilot test of the HOPE intervention to explore employment and mental health among African American gay men living with HIV/AIDS: results from a CBPR study | Hergenrather (2013) | African American MSM | United States | Prospective cohort, pre-post | Mental health, employment |
| Lifestyle Intervention for Filipino Americans at Risk for Diabetes | Inouye (2014) | Filipino Americans | Hawaii, United States | Randomized wait list controlled trial | Reduce risk of Diabetes |
| Promoting Enrollment in Parenting Programs Among a Filipino Population: A Randomized Trial | Javier (2019) | Filipino Americans | California, United States | Randomized Controlled Trial, parallel | Parenting, Adolescent Health |
| Cultural Dance Program Improves Hypertension Management for Native Hawaiians and Pacific Islanders: a Pilot Randomized Trial | Kaholokula (2015) | Native Hawaiians and Pacific Islanders | Hawaii, United States | Randomized Controlled Trial | Physical Activity, Hypertension |
| The power of women’s and men’s Social Networks to catalyse normative and behavioural change: evaluation of an intervention addressing Unmet need for Family Planning in Benin | Kim (2022) | Adults | Benin | Cross-sectional, pre-post | Contraceptive use |
| Development and feasibility of a childhood obesity prevention program for rural families: application of the social cognitive theory | Knol (2016) | Rural, low-income families | Southern United States | Feasibility, pre-post | Sedentary behaviors, eating patterns, nutrition |
| The Health Equilibrium Initiative-Is it Possible to Prevent Intervention- Generated Inequality? | M (2017) | Low income, immigrant families and children | Sweden | Repeated cross-sectional and longitudinal | Overweight, obesity in children |
| Increasing Cervical Cancer Screening Among Vietnamese Americans: A Community-Based Intervention Trial | Ma (2015) | Vietnamese Women | Eastern United States | Randomized Controlled trial | Cervical cancer screening |
| Interventional Audiology to Address Hearing Health Care Disparities: Oyendo Bien Pilot Study | Marrone (2017) | Older adults in Rural Arizona | Arizona, United States | Pilot group-based intervention, pre-post | Hearing health |
| A community-based, environmental chronic disease prevention intervention to improve healthy eating psychosocial factors and behaviors in indigenous populations in the Canadian Arctic | Mead (2013) | Indigenous populations | Canada | Quasi-experimental, pre-post | Chronic diseases, healthy eating |
| Kids Identifying and Defeating Stroke (KIDS): Development and Implementation of a Multiethnic Health Education Intervention to Increase Stroke Awareness Among Middle School Students and Their Parents | Mullen Conley (2010) | Mexican-American children and their families | Texas, United States | Randomized Controlled Trial | Stroke knowledge |
| Evidence-based intervention program for reducing obesity among African-American women in Southern California | Onyegbule (2021) | African American Women | California, United States | Quasi-experimental | Weight loss |
| Impact of a community-based prevention marketing intervention to promote physical activity among middle-aged women | Sharpe (2010) | Women aged 35-54 | Southeastern United States | Nonrandomized cross-sectional | Physical Activity |
| Measuring our success in teaching Latinos about asthma and home environments: lessons learned from an intervention developed through photovoice | Trujillo (2020) | Latino parents | United States | 1-arm, pre-post | Reduce risk of asthma |
| A social network family-focused intervention to promote smoking cessation in Chinese and Vietnamese American male smokers: a feasibility study | Tsoh (2015) | Vietnamese American Male Smokers | United States | 1-arm, pilot | Smoking cessation |
| The iCook 4-H Study: an intervention and dissemination test of a youth/adult out-of-school program | White (2019) | Youth ages 9-10 | United States | Randomized Controlled Trial | Obesity prevention |
| Improving immunization rates through community-based participatory research: community health improvement for Milwaukee's children program | Willis (2016) | Black and Hispanic children and families | Milwaukee, United States | Cross-sectional, pre-post | Vaccination |
