# Supplementary material for: Applications of equity frameworks in theory-based health behavior interventions: a scoping review
Source: Int J Equity Health. 2025 Mar 20;24:79. doi: 10.1186/s12939-025-02438-x (PMC11924764; doi:10.1186/s12939-025-02438-x)
Supplement: Supplementary file 3 — Supplementary Material 3. [file 12939_2025_2438_MOESM3_ESM.docx]

**Supplementary Table 3: Data extractions of theories, equity frameworks and results from review of studies (n=26)**

| Title | Author (Year) | Theory Applied | Equity Framework Applied | Intervention Design & Components | Results (brief) | Description of intervention development & Integration of theory and equity framework | Theme |
| --- | --- | --- | --- | --- | --- | --- | --- |
| A pilot test of a church-based intervention to promote multiple cancer-screening behaviors among Latinas | Allen (2014) | - Integrative Model of Behavioral Prediction | - Community-Based Participatory Research - Socio-Ecological model | - Design: One-group pre-/post-evaluation within a low-income, Latino Baptist church (n=35) - Components: 6-month church-based education program | 24% increase in adherence with breast cancer screening recommendations; not statistically significant.  Intervention was feasible with high acceptability  Participation rates: charlas (72%), health fairs (61%), goal-setting campaign (50%) | - Theory informed intervention - Constructs were measured as outcomes - Evidence-based strategies selected based on recs from the Community Preventive Services Task Force - Materials gathered from Research Tested Intervention Programs (RTIPS) and Cancer Control P.L.A.N.E.T. - Standardized process for adapting existing materials for the audience and the community context - Process overseen by investigators working with a Community Advisory Board (CAB) - Intervention was implemented by Peer Health Advisors (PHAs) | Constructs informed activities and design  Constructs measured as outcomes  Use of Community Advisory Board (3)  Standardized process for integrating feedback  Use of Community Health Workers (delivery) |
| "We are a powerful movement”: evaluation of an endometrial cancer education program for black women | Alson (2021) | - Health Belief Model | - Community-Based Participatory Research - Public Health Critical Race Praxis | - Design: Pre-post cancer education program (n=62) - Components: Ambassador training (n=13). Ambassador-led education sessions & use of clinical self-advocacy tools | Non-significant increase in ambassador knowledge. Participant knowledge increased (2.02 points (95% CI, 1.06–2.99; p = 0.0001))  Intervention was feasible and acceptable | - Constructs of HBM directly informed intervention activities - Used Public Health Critical Race Praxis principles of Voice, Primacy of Racialization, Critical Approaches, and Intersectionality in quantitative and qualitative methods - Adaptation of Community Empowerment Partners for Endometrial Cancer (CEPs-EC) intervention for a nationwide implementation - Evaluation is a project of the Endometrial Cancer Action Network for African Americans - History of partnership - Steering group includes researchers and one survivor/patient - Quantitative survey instrument was designed with the full participation of the CEPs-EC team - Adaptation of educational materials occurred in an iterative process to ensure team agreement. - Compensation for participant’s time - Mentorship based on trust and the use of social networks contributed to feasibility - Ambassadors without personal connections to Black women faced challenges recruiting participants | Constructs informed activities and design  Established history of partnership, trust *(and lack thereof)*  Use of Community Advisory Board (3)  Participant involvement in research (evaluation tools)  Standardized process for integrating feedback  Principles and process for sharing power (decision making)  Accommodations (compensation) |
| "No Queremos Quedar Mal": A Qualitative Analysis of a Boundary Setting Training Among Latina Community Health Workers | Alvarez-Hernandez (2021) | - Social Cognitive Theory | - Chicana feminist framework - Community-based participatory research | - Design: Observational, cohort, qualitative - Components: Four 1-hour boundary-setting training sessions over 3 months for CHWs (n=5) - Evaluation at Y1 | Training had a positive effect on participant’s sense of professionalism, emotional well-being, and ability to establish boundaries. | - Theory: Training considered the constructs related to personal cognitive factors, socio-environmental factors, and behavioral factors - Chicana feminist framework used to critically consider gendered and social class norms among people from Mexican origin - CBPR guided the development of gender- and culturally-responsive training - Following Chicana feminism, the authors linked participants’ statements about their experiences as promotoras to gendered sociocultural scripts for Latina women - Insights guided the development of gender- and culturally-responsive training - Negotiating gendered and cultural expectations with boundary setting - Materials in the native language | Constructs informed activities and design  Critical reflection of inequities (social norms root causes)  Materials, activities, content reflect social norms and values (cultural relevance)  Standardized process for integrating feedback  Materials available in 2+ languages |
| Feasibility and Outcomes of an HIV Testing Intervention in African American Churches | Berkley-Patton (2019) | - Theory of planned behavior | - Community-based participatory research - Socio-Ecological model | - Design: Cluster Randomized Controlled Trial (n=543) - Components: Church-based HIV testing over 12 months - Individual, group, church-wide, community activities | Significant increases in HIV testing (59% vs. 42% at 12 months p = 0.008), and particularly church-based testing (54% vs. 15%, p < 0.001), relative to controls. Intervention group received testing at rate 2x faster than controls. Increases in Intention and beliefs. No reduction in social normative support or HIV stigma. | - Intervention materials and activities were directly informed by TPB constructs, which were measured in the study outcomes - CBPR: Faith leaders and agencies were engaged in all phases of the research process - Socio-ecological model: intervention was designed using a multi-level approach to fit naturally into existing community norms and spaces - Faith leader contributions include scriptural references in materials & review of materials - Use of Church Health Liaisons (CHL) for event coordination | Constructs informed activities and design  Constructs measured as outcomes  Use of Community Advisory Board (4)  Multi-level approach (individual, interpersonal, community)  Use of Community Health Workers (coordination)  Use of Community Members, influential leaders (ambassadors) |
| The development of an innovative, theory-driven, psychoeducational HIV/STI prevention intervention for heterosexually active Black adolescents with mental illnesses | Brawner (2019) | - Theory of Planned Behavior | - Social determinants of health framework | - Design: Pilot randomized controlled trial (n=108) - Components: Two-day program with 8 45-inute modules on HIV prevention strategies | No statistically significant differences observed in behavior change (condom use, # sexual partners) from baseline. Lower rates of depressive symptoms were found in intervention group at 3-month follow up. | - TPB constructs guided intervention activities and were measured as outcomes - SDOH framework directly informed intervention activities through consideration of social factors around racial identity, gender norms, poverty, education, and sexual activity – intersectionality - Community Advisory board provided study oversight comprised of 8 youth who aided in study design, implementation, and evaluation - Use of Non-traditional, multi-level approach - Specific examples of how feedback from CAB directly informed materials - Compensation provided - Community members as facilitators - 2-year planning process - Blackness as a source of resilience, pride, and unspoken expectations in sexual relationships emerged as themes | Constructs informed activities and design  Constructs measured as outcomes  Multi-level approach (individual, interpersonal, community)  Critical reflection of inequities (intersectionality, social norms)  Use of Community Advisory Board (3)  Standardized process for integrating feedback  Accommodations (compensation)  Use of Community Members, influential leaders (CM facilitators)  Established history of partnership (time intensive) |
| Group Versus Individual Culturally Tailored and Theory-Based Education to Promote Cervical Cancer Screening Among the Underserved Hispanics: A Cluster Randomized Trial | Calderón-Mora (2020) | - Health Belief Model - Theory of reasoned action - Social cognitive theory | - Socio-ecological Model - Intervention mapping & Community Based Participatory Research | - Design: Cluster Randomized Controlled Trial (n=300) - Components: Group and individual health education on cervical cancer screening plus navigation services and free testing. | Group education was as effective as individual education in increasing the uptake of cervical cancer screening. Constructs perceived benefits, self-efficacy, perceived susceptibility, perceived seriousness increased. | - Theoretical constructs measured through the surveys included items from the Health Belief Model (HBM), Theory of Reasoned Action (TRA), and Social Cognitive Theory (SCT). - Table outlines the AMIGAS Intervention Determinants, Methods and Strategies - Intervention targeted multiple levels of the socioecological model including individual, interpersonal, and organizational levels (health care system) to address structural barriers - IM was used in the context of a community-based participatory research (CBPR) approach to develop a culturally appropriate, theoretically sound intervention for Hispanic women - Use of promotoras - Use of Community Advisory Board included practitioners from the community, community-based organizations, CHWs on other projects, and community members - CAB role was to oversee research and to make decisions about methods, goals, materials, recruitment, and how funds are spent - Not involved data collection/analysis - Discussion of theoretical constructs with CAB - Thorough in description of how CAB feedback was implemented | Constructs measured as outcomes  Constructs mapped to intervention activities  Participant involvement in research (understanding theory, not evaluation tools)  Multi-level approach (individual, interpersonal, community)  Materials, activities, content reflect social norms and values (cultural relevance)  Principles and process for sharing power (decision making, resource allocation)  Use of Community Advisory Board (4)  Standardized process for integrating feedback  Use of Community Health Workers (coordination, delivery, facilitation) |
| Group Dance and Motivational Coaching for Walking: A Physical Activity Program for South Asian Indian Immigrant Women Residing in the United States | Daniel (2021) | Social cognitive theory | - The Physical Activity Framework for South Asian Indian Immigrants - Cox’s Interaction Model of Client Health Behavior | - Design: 2-arm Intervention (n=50) - Components: Biweekly physical activity workshops for 24 weeks. Motivational workshops, dance, follow up calls | No significant differences in body weight loss between groups. Increases in self-efficacy, | - Motivational workshops developed based on Social Cognitive Theory; constructs were measured as outcomes - Trust building and networking with SAI community stakeholders was established to support recruitment of participants - Cox’s Model used to understand population background characteristics (demographic, social influences, discrimination, social support) influence interpersonal and health behavior - Intervention focus was on social supports; - PI and researchers were bilingual and of the same race/ethnicity as participants | Constructs measured as outcomes  Critical reflection of inequities (oppressive systems, social norms)  Multi-level approach (individual, interpersonal)  Researchers shared characteristics of population (language, ethnicity)  Established history of partnership, trust, time intensive |
| Educating Hispanic Women about Cervical Cancer Prevention: Feasibility of a Promotora-Led Charla Intervention in a Farmworker Community | Fleming (2018) | - Social Cognitive Theory - Health Belief Model | - Community Based Participatory Research | - Design: 1-Arm Pretest/Posttest Design (n=60) - Components: Health ambassadors facilitate health education programming - Follow up at 3 months | Significant increase in HPV knowledge and Pap test self-efficacy (F[1,59]>22.7, Ps< .0001) but not for health beliefs (F[1,59]=.49, P=.48). No significant change in the intention to have a Pap test within the next 6 months (M=.5, P=.99) | - The constructs of the SCT and HBM were carefully and systematically mapped to the content of the curriculum - Curriculum drew from an existing cervical cancer curriculum and educational resource (CCER) developed using CBPR in a neighboring community - Promotoras were highly trusted Hispanic women who were familiar with the local community culture and values and had an interest in health improvement for their community. - Existing partnership with community groups - Partnership is formally defined in a memorandum of understanding and involves a mutually beneficial exchange of expertise and resources - A lay bilingual patient navigator hired as staff - Patient navigator was instrumental in the creation of this educational intervention and ensured its cultural and linguistic relevance - Promotoras informed curriculum CBPR | Constructs mapped directly to intervention activities  Constructs measured as outcomes  Use of Community Health Workers (informants, ambassadors)  Established history of partnership  Partnership with community is formalized (MOU)  Materials, activities, content reflect social norms and values (cultural relevance)  Researchers shared characteristics of population (language) |
| Dash of Faith: A Faith-Based Participatory Research Pilot Study | Harmon (2014) | - Social Cognitive Theory | - Community Based Participatory Research | - Design: Quasi-experimental (n=23) - Components: Experiential dietary intervention based in two African-American churches. Weekly classes for 12 weeks + 4 monthly classes for 8 months | **F&V intake**: initial increase in F&V consumption among intervention group was marginally significant and  not sustained at 8-month follow up.  **Fat intake**: The control group had a greater reduction of fat intake.  **Body Weight**: both groups increased body weight. | - Aspects of SCT such as goal setting and the inclusion of church leaders who serve as role models also were included in the intervention design - Community Advisory Panel of 10 community leaders was created to determine the content, format, and implementation procedures of the intervention - Project was requested from the community - Class content was directed by intervention participants | Constructs informed activities and design  Use of Community Advisory Board (4)  Community-identified priorities  Standardized process for integrating feedback  Materials, activities, content reflect social norms and values (cultural relevance) |
| A pilot test of the HOPE intervention to explore employment and mental health among African American gay men living with HIV/AIDS: results from a CBPR study | Hergenrather (2013) | - Social Cognitive Theory - Hope Theory | - Community Based Participatory Research | - Design: Prospective cohort, pre-post with 3 month follow up (n=7) - Components: 7 weekly 3-hour group sessions | At 3-month post-intervention: significant improvements in mental health outcomes (anxiety and depression), medication adherence, communication with healthcare providers, self-efficacy related to job-seeking and coping self-efficacy | - 10-year history of partnership with local community including prior studies - Partnership includes community health center, academic institutions, and members of target population (AA gay men) - CBPR partnership reviewed and disused behavioral theory to understand how interventions could promote positive behavior change - Integrated theory with perspectives on African American gay men’s lived experiences to make informed decisions about the intervention and establish co-ownership. - Constructs directly informed intervention activities and were measured as outcomes. - Thorough participation by CPBR members, including AA gay men, in priority-setting, literature review, goal setting, intervention development, discussion of behavior theory - Researchers shared race/ethnicity with participants and also identified as gay - Firm adherence to CBPR principles - Use of photovoice as human-centered design method - Barriers identified at several levels of the SEM | Constructs informed activities  Constructs were measured as outcomes  Use of Community Advisory Board (5)  Researchers shared characteristics with population  Participant involvement in research methods (understanding theory, literature review)  Critical reflection of inequities  Multi-level approach (individual, interpersonal, community)  Established history of partnership (prior studies)  Community-identified priorities  Principles and process for sharing power (transparency, decision making, conflict resolution)  Co-learning mindset (co-leading, co-owning models) |
| Lifestyle Intervention for Filipino Americans at Risk for Diabetes | Inouye (2014) | - Social Learning Theory | - Community Based Participatory Research | - Design: randomized wait-list control design (n=40) - Components: Eight-session curriculum delivered over 6 months. Presentations and diabetes screening. | Weight loss and waist-reduction were statistically significant in the intervention group. Reduced diabetes risk factors by 24%. High retention rate of 88%. | - SLT constructs directly informed intervention activities and were measured as outcomes. - The Philippine Nurses Association (PNA) of Hawaii acted as the research partner in implementation of participatory research and identifying diabetes prevention as a priority - Study was designed to fill a gap in research on diabetes prevention interventions tailored to Filipino cultural themes - History of partnership between principal investigators and the Filipino community over the 3 years prior to the intervention. - Eight sessions from the DPP interventions were culturally tailored to include diet and exercise options more commonly used by Filipinos. - Facilitator of the intervention was fluent in English and two Filipino dialects; small groups were led by a Filipino healthcare worker - Focus groups with Filipino parents with children at home were conducted, as well as a review of the curriculum by the PNA Advisory group which consisted of 3 PNA board members. - Flexible scheduling of the curriculum on weekends to accommodate working parents | Constructs informed activities and design  Constructs measured as outcomes  Established history of partnership  Community-identified priorities  Materials, activities, content reflect social norms and values (cultural relevance)  Researchers shared characteristics of population (language, ethnicity)  Use of Community Advisory Board (3)  Accommodations (scheduling) |
| Promoting Enrollment in Parenting Programs Among a Filipino Population: A Randomized Trial | Javier (2019) | - Health Belief Model - Theory of Planned Behavior | - Community Based Participatory Research | - Design: 2-group parallel, randomized trial design with pre-post follow up - Components: Educational video | Significantly higher knowledge of Filipino adolescent behavioral health disparities and higher perceived susceptibility to adolescent risky sexual activity and illegal drug use. Intervention group was more likely to enroll in the parenting class. | - Video included cues to influence constructs of the Health Belief Model and Theory of Planned Behavior - TBP and HBM constructs measured as outcomes - Constructs map directly to activities: Modifiable Predictors of Participation and Corresponding Engagement Strategies - Researchers map theoretical constructs to culturally relevant intervention strategies - Community Advisory Board (CAB) consisted of Filipino parents, community-based organization leaders, educators, health and mental health providers, parents, and grandparents. - CBPR partnership discussed literature and drove content decisions, messages, media, related to the educational video - Researchers had history with Filipino community stakeholders dating back five years - Researchers provided thorough descriptions of how intervention was adapted based on cultural findings: several qualitative studies were conducted. - The decision to include both mothers and fathers and a grandmother and to include a mix of Tagalog messages with English subtitles were suggestions made by our CAB and community stakeholders - Culturally relevant components include an all-Filipinos “cast” in the video, content delivered in English and Tagalog, and emphasis on familial dynamics and values within Filipino communities. - Discussions on colonialism and fatalistic views highlighted Filipinos common resistance to colonizers - Researchers state this is the first theory-based education video that seeks to motivate Filipinos to take action and prevent adolescent risky behaviors | Materials, activities, content reflect social norms and values (cultural relevance)  Constructs informed activities and design  Constructs measured as outcomes  Constructs map to interventiona activities  Direct link between constructs and culturally-relevant content  Standardized process for integrating feedback  Use of Community Advisory Board (5)  Multi-level approach (individual, interpersonal, community, society)  Critical reflection of inequities (oppressive systems)  "First of its kind" intervention  Established history of partnership (trust, time intensive, prior studies) |
| Cultural Dance Program Improves Hypertension Management for Native Hawaiians and Pacific Islanders: a Pilot Randomized Trial | Kaholokula (2017) | - Social Cognitive Theory | - Community Based Participatory Research | - Design: 2-Arm Randomized Controlled Trial (n=27) - Components: 12-week hula-based pilot delivered in a community health center. Two 60-minute sessions included hypertension education and dance classes. | Intervention group had greater reductions in SBP than wait-list control participants. No significant differences in DBP, physical functioning, or HRQOL. Retention was high at 87%. | - Intervention design was informed by SCT. - Researchers’ ties to the indigenous, patient, and medical communities helped to attain the networks and community support needed for the development and implementation of the intervention. - Community Investigators included a kumu hula (hula expert) and two NHPI community leaders who partnered with a Native Hawaiian scientist with extensive experience undertaking research in Native Hawaiians communities. - Researchers detail how clinical and community recommendations led to adaptations of the intervention: table titled “Selected Cultural Recommendations from Focus Groups and Application to Intervention Design” - An example of how integration of cultural and clinical information informed the development of the program was intervention staffing. The cultural and patient community viewed credentials for the instructor of hula important for authenticity and credibility - Curriculum was previously developed and culturally adapted for NHPI based on the Heart Failure Society of America’s educational guidelines and modules - Focus group sessions are congruent with the Native Hawaiian tradition of storytelling and oral history and provided an open-ended venue for participants to share their opinions and thoughts. - Community Health Workers facilitated sessions - Content was consistent with CBPR principles and Native Hawaiian cultural protocol including cooking demonstrations using NHPI ethnic foods, and the involvement of elders as key informants - Allowing time for a thorough and balanced discussion that was inclusive of investigators, scientific–clinical, and community–cultural expertise, a more comprehensive understanding of objectives for CR and for hula education was communicated. | Constructs informed activities and design  Researchers shared characteristics of population (language, ethnicity)  Use of Community Members, influential leaders (ambassadors, facilitators)  Standardized process for integrating feedback  Materials, activities, content reflect social norms and values (cultural relevance)  Use of Community Health Workers (facilitation)  Culturally-relevant content  Multi-level approach (individual, interpersonal, community)  Critical reflection of social norms, root causes  Principles and process for sharing power (transparency, decision making, conflict resolution) |
| The power of women’s and men’s Social Networks to catalyse normative and behavioural change: evaluation of an intervention addressing Unmet need for Family Planning in Benin | Kim (2022) | - Social Network Theory | - Framework to reduce socio-formative barriers - Social Network Mapping | - Design: Pre-post cross sectional study (n=2091) - Components: Social network mapping, critical reflection, use of influential network actors to promote family planning, social marketing, and links to family planning services. | Significant improvements in intention to use contraception and met need.  Women’s use of contraception increased however results were not statistically significant. | - Intervention approach was directly informed by Social Network Theory, using communication as a social change technique. - Theory of Change includes components at multiple levels of the socio-ecological model - Researchers facilitated a critical review of the literature on social network initiatives, unclear how findings were shared with the community or participants - Community groups participate in mapping exercises to identify the most influential and connected network actors in their villages - Social groups and opinion leaders (influential network actors) engage women and men in participatory, critical reflection-style dialogues to promote learning, diffusion of new ideas, and discuss fertility concerns and taboos - Goal of critical dialogue is broader socio-normative change - Materials designed to reflect on social norms - Processes are put in place to link community members with health services | Constructs informed activities and design  Constructs measured as outcomes  Use of Community Members, influential leaders (ambassadors, facilitators)  Materials, activities, content reflect social norms and values (cultural relevance)  Multi-level approach (individual, interpersonal, community, societal)  Critical reflection of inequities (oppressive systems, social norms)  Use of Community Advisory Board (5) |
| Development and feasibility of a childhood obesity prevention program for rural families: application of the social cognitive theory | Knol (2016) | - Social Cognitive Theory | - Community-Based Participatory Research - Home Food Environment Model of Childhood Obesity | - Design: Feasibility study, pre-post test (n=34) - Components: Five educational sessions on mindful eating; gamification, group discussion; parent-child activities | Three of the four target behaviors improved over the intervention period. Hours of TV watched and consumption of “red light” foods decreased. Mindful eating was the only personal construct that significantly improved. | - SCT constructs were embedded into the curriculum and measured as outcomes. - Community collaborators were involved in all aspects of program development and implementation to ensure that each aspect of the program was culturally appropriate - No further detail on application of CBPR principles - Researchers map the program’s components to SCT constructs - Interdisciplinary research team consisted of a dietitian, three community collaborators, a health educator, and two psychologists (one specifically trained in mindfulness) developed the HSH program. | Use of Community Advisory Board (3)  Constructs mapped directly to intervention activities  Multi-level approach (individual, interpersonal) |
| The Health Equilibrium Initiative-Is it Possible to Prevent Intervention- Generated Inequality? | M (2017) | - Social Cognitive Theory | - Community-Based Participatory Research - Intervention mapping | - Design: Repeated cross-sectional and longitudinal (n=119) - Components: School-based campaign (lessons, dialogues, lectures) to promote healthy behaviors and physical activity. Attempts to influence structural level-factors related to healthy schools. | No significant difference in BMI, food patterns, or SCT constructs between children in control and intervention schools. | - Activities were designed to influence Social Cognitive Theory (SCT) constructs, which were measured as outcomes - Researchers coordinated with dieticians, parent groups, school nurses, and other school-based organizations to build support for addressing the health gap. - Structure and specific goals of intervention activities (“constructed dialogues”) are lacking - Cited “short term” nature of the intervention (1 year) as problematic - Unclear how CBPR principles were applied, despite researchers stating that processes were thoroughly documented. - HEI was involved in 58 meetings with school staff; 7 meetings in the parental group and 12 CBO group. - Several activities were described as “initiated” and later abandoned with no rationale - Intervention was welcomes initially by schools but lacked in continuity regarding support and commitment - Quantitative portion of the intervention was planned beforehand and therefore did not involve participants; no efforts to build trust ahead of intervention. Researchers cite funding as reason for this. - One family, in their refusal to participate, shared a note with researchers expressing a strong lack of trust and confidence - Researchers tried with no success to build meaningful relationships with school staff in early stages. | Constructs informed activities and design  Constructs measured as outcomes  Persistent, sustainability, continuity issues  Established history of partnership (lack thereof, trust specifically) |
| Increasing Cervical Cancer Screening Among Vietnamese Americans: A Community-Based Intervention Trial | Ma (2015) | - Health Belief Model - Social Cognitive Theory | - Community-Based Participatory Research | - Design: Two-arm Randomized Controlled Trial (n=1,488) - Components: Sessions led by community health educators referral to screenings, reminders - Outcomes measured at immediately post and 12-month follow up | Statistically significant increase in Pap testing among intervention group than control group (p< .0001) | - Multifaceted intervention included community health educator (CHE)-led group education culturally relevant visual aids, and patient navigation - One of the first largest randomized community-based intervention trials, aimed to increase cervical cancer screening and reduce multilevel barriers among medically underserved and low-income Vietnamese women - 42 Vietnamese community leaders were directly involved in the planning, development, and implementation of the project through planning and advisory groups - Addressed both individual choices and health care system barriers through multifaceted innovative program elements - Vietnamese Community organizations provided navigation in language (e.g. bilingual assistance available) and sociocultural barriers - Findings demonstrated that using a CBPR approach is more likely to increase the intervention effect on the receipt of Pap tests and the sustainability of an intervention research program. - HMB and SCT constructs were measured as outcomes | Multi-level approach (Individual, interpersonal, community)  Use of Community Health Workers (facilitation)  "First of its kind" intervention  Use of Community Advisory Board (4)  Materials, activities, content reflect social norms and values (cultural relevance)  Critical reflection of inequities (social norms)  Constructs measured as outcomes  Materials available in 2+ languages |
| Interventional Audiology to Address Hearing Health Care Disparities: Oyendo Bien Pilot Study | Marrone (2017) | - Health Belief Model | - Community-Based Participatory Research | - Design: Single-arm, group-based intervention (n=21) - Component: 5-week, Spanish-language hearing health education program for older adult with qualitative evaluation | Post-program focus groups revealed increased self-efficacy and decreased stigma. Combinations of perceived severity and self-efficacy catalyzed action. After 1 year, 7 of 9 participants with hearing loss contacted for follow-up had sought some form of hearing-related health care. | - Facilitated through an academic-community partnership between audiology, public health, and community health workers of a Federally-Qualified Health Center, with whom researchers had a long-standing relationship. - Rationale for the intervention was to develop and evaluate of an audiology approach that supports goals for health equity and reduced disparities in access to hearing care. - CHW knowledge of their border community informed the development and cultural adaptation of a hearing health outreach program to be culturally and linguistically relevant for Spanish-speaking Hispanic/Latino older adults - CHWs were uniquely positioned to engage community members in research as trusted members of and trained health educators. - CHWs at the FQHC received specialized training on hearing loss and how to communicate effectively with individuals and families with hearing loss - Conducted a needs assessment based on the Health Belief Model to gain understanding of health beliefs related to hearing loss and access to care in a rural, Hispanic/Latino community. - Provided free hearing screening as a first step in developing relationships within the community around hearing loss - Qualitative data was coded from the needs assessment and post-program focus groups based on constructs of the HBM - Activities were tailored for appropriateness in the cultural, rural, and FQHC contexts based on input from the community partners and content was adapted for facilitation by nonclinical health educators (CHWs) - Persons with hearing loss and family members expressed the importance of Mexican cultural values related to the development and implementation of a potential hearing health program | Established history of partnership (trust, time intensive, prior studies)  Multi-level approach (individual, interpersonal, community)  Critical reflection structural influences (oppressive systems, social norms)  Use of Community Health Workers (informants, coordination, delivery, facilitation)  Researchers shared characteristics of population (language, ethnicity)  Materials, activities, content reflect social norms and values (cultural relevance)  Constructs informed activities and design  Use of Community Advisory Board (4)  Materials available in 2+ languages |
| A community-based, environmental chronic disease prevention intervention to improve healthy eating psychosocial factors and behaviors in indigenous populations in the Canadian Arctic | Mead (2013) | - Social Cognitive Theory - Theory of Planned Behavior | - Socio-ecological models - Community-Based Participatory Research | - Design: Quasi-experimental pre-post-evaluation at 1-month follow up - Components: 12-month, multi-level intervention included events, media, health information sessions & demonstrations - 6 remote communities received intervention; 2 matched control communities | Improvements in self-efficacy (p = .003) and intentions (p = .001) in intervention group. Decrease in overweight, obese, healthy food consumption and purchase of unhealthy foods. Improvements in self-efficacy and intentions to eat healthy foods. No impact on BMI. | - Large and diverse stakeholders were partners: local stores, national food retailers, a local research institute, local and national governmental organizations, and multiple levels of government - The process to inform the 12-month intervention was 4 years prior to the start of preintervention data collection. - Community members participated in workshops to develop the intervention and materials (key messages, promotional strategies, and the selection of healthier foods, beverages, and preparation methods to promote) - Community collaboration and ownership continued throughout the intervention through regular presentation of research results and study progress to the stakeholders for feedback. - Cultural norms and values were captured through formative research and incorporated into the intervention. - Community members were hired and trained to deliver the intervention and offer feedback on design - The intervention worked on an environmental level with local food stores, retailers, and other partners to increase the availability and accessibility of healthier food options and opportunities for engaging in physical activity - Activities designed around the theoretical constructs of SCT. - Community-level components includes radio and TV media and community-wide activities in recreational centers, health and wellness centers, worksites, schools, and other venues. - Data collectors fluent in the local language conducted the interview or an interpreter was used. | Use of Community Advisory Board (5)  Accommodations for participants (compensation)  Multi-level approach (Individual, interpersonal, community)  Constructs informed activities and design  Constructs measured as outcomes  Researchers shared characteristics of population (language)  Established history of partnership (time intensive,  Standardized process for integrating feedback  Principles and process for sharing power (transparency, decision making)  Co-learning mindset (co-leading, co-owning models)  Use of Community Members, influential leaders (delivery)  Materials, activities, content reflect social norms and values (cultural relevance) |
| Kids Identifying and Defeating Stroke (KIDS): Development and Implementation of a Multiethnic Health Education Intervention to Increase Stroke Awareness Among Middle School Students and Their Parents | Mullen Conley (2010) | - Social Cognitive Theory |  | - Design: Prospective, randomized, controlled school-based trial with pre-interim tests. - Components: four one-hour classes on stroke education for kids per year plus homework assignments with parents | Successful in improving students’ stroke symptom and treatment knowledge and intent to call 911 upon witnessing a stroke compared with controls (p<0.001) | - KIDS Planning Committee met weekly to discuss the lessons as they were designed and implemented - Community surveys in East Texas and in Corpus Christi provided insights into stroke related SCT constructs - KIDS incorporated MA values of familisms and social norm of multi-generational households - Mexican American (MA) health professional developed lessons with focus groups with parents, teachers, and students who provided feedback on cultural relevance - Authors provide specifics on how feedback was used to culturally tailor the intervention - the CHWs facilitated a bridge between the researchers and community, which improved communication and contact with underserved families. - A representative example of familismo, or placing the family's needs over the individual's needs, included many family members pitching in to buy a hearing aid. - Materials available in both English and Spanish - SCT constructs Behavioral capability, self-efficacy, and outcome expectations received special emphasis and were measured as outcomes - Materials included geographies, landmarks, and cultural references familiar to MA children | Use of Community Advisory Board (4)  Critical reflections of social norms  Researchers shared characteristics with population  Standardized process for integrating feedback  Constructs informed activities  Constructs were measured as outcomes  Materials, activities, content reflect social norms and values (cultural relevance)  Materials available in 2+ languages |
| Evidence-based intervention program for reducing obesity among African-American women in Southern California | Onyegbule (2021) | - Health Belief Model | - Empowerment model | - Design: Quasi-experimental (n=28) - Components: Health education and weight-loss management program with support groups and empowerment-building | The results showed that 90% of participants lost 20 pounds or more and 82% had 6 inches or more waist circumference reductions. Cholesterol reductions and improved nutrition knowledge and exercises were significant. | - Focus groups provided insight on HBM constructs which informed intervention design and cultural tailoring - Empowerment model activities included motivational speaking and posters of non-obese AAW in exam rooms - Primary investigator was an AAW doctoral NP, who facilitated group discussions - No further detail on how the empowerment model was applied - No further detail on methods for cultural tailoring - Free transportation provided and fresh vegetables donated and delivered to participant’s homes; childcare provided - Intervention is largely focused on changes to individual behaviors | Constructs informed activities and design  Accommodations, compensation for participants  Single-level intervention  Researchers shared characteristics of population (ethnicity) |
| Impact of a community-based prevention marketing intervention to promote physical activity among middle-aged women | Sharpe (2010) | - Social Cognitive Theory | - Community-Based participatory research - Community- Based prevention Marketing Framework | - Design: Nonrandomized cross-sectional design (n=430) - Components: 24-week physical activity program (n=430) and year-long media campaign | Intervention group had significant positive increases on several physical activity behaviors and the media campaign demonstrated increase in knowledge of PA routes. Small average weight loss for intervention group | - CBPR partnership extends 5 years prior to the social marketing intervention - CBPR partnership rooted in environmental change: “active community environment” through changes in the community environment and related policies.” - CBPR and CBPM principles guided the intervention development process - SCT informed the design of specific intervention materials, messages, and strategies. - Funding for the intervention came through an existing Community Advisory Board (CAB) partnership to expand ongoing work and address more levels of influence on behavior - The intervention combined behavioral theory with principles of social marketing within a CBPR framework, called community-based prevention marketing - CAB included experienced coalition members and new members with a specific interest in physical activity; 40 CAB members and community spokespersons included AA women, 2 AA men, 20 White women, and 3 White men - CAB members attended a workshop on social marketing framework and helped develop questionnaires - Meetings were tape-recorded, meeting minutes distributed and findings from formative research were presented to CAB members who helped organize findings - Protocol was established for managing conflict - CAB members provided feedback on experience with CBPR process - Guidance on sociocultural issues came from the formative research as well as the CAB members’ input - Community-driven preferences and profiles, along with theory-based principles, guided the development and tailoring of all messages, materials, and activities to address women’s preferences and perceptions. | Established history of partnership (trust, time intensive, prior studies)  Multi-level intervention (individual, interpersonal, community)  Use of Community Advisory Board (5)  Constructs informed activities and design  Constructs measured as outcomes  Participant involvement in research/methods (evaluation tools, understanding theory)  Principles and process for sharing power (transparency, decision making, conflict resolution, shared resources)  Adherence to CPBR evaluated by CAB  Critical reflection of structural factors (social norms)  Community-identified priorities  Materials, activities, content reflect social norms and values (cultural relevance) |
| Measuring our success in teaching Latinos about asthma and home environments: lessons learned from an intervention developed through photovoice | Trujillo (2020) | - Social Cognitive Theory | - Community-based participatory research - Photovoice | - Design: One-group pre-post test design (n=19) - Components: 60-minute group education program and 2-hour educational intervention | Evaluation produced mixed results. Self-efficacy increased for mitigating home environments and tobacco exposures. Knowledge decreased on controlling mold and indoor triggers declined. | - CBPR project led by Latina mothers, with support from a bilingual cultural liaison, an interpreter, and a research support team (RST) - Cultural liaison, who had a background as a Community Health Worker promoting asthma control, helped coordinate and facilitate meetings - RST coordinated development of the intervention, provided supports for strengthening the partnership, and designed the data collection tools with input from the women - Lesson plans were developed around a culturally appropriate practice known in Mexico as demostración (discussion) circles - The interpreter provided simultaneous interpretation during meetings and translated our materials and study tools. - Mexican-American mothers in the team participated as peer educators in the intervention - All members of the project were involved in describing the problem, analyzing data, discussing findings and implications, and revisions of the manuscript - The grant provided reimbursement ($50/month) for the Mexican-American women members - Researchers (community members) felt they lacked skilled in evaluation design - Process of developing and testing instruments using CBPR principles required significant time, facilitation, and training - Differences in conceptual understandings of surveys between the interpreter and the peer educators who, while both Spanish-speakers, were from different parts of Mexico - Limited detail on application of Social Cognitive Theory | Use of Community Advisory Board (5)  Use of Community Health Workers (facilitators)  Materials, activities, content reflect social norms and values (cultural relevance)  Co-learning mindset (co-leading, co-owning models)  Accommodations, compensation  Use of Community Members, influential leaders (ambassadors, facilitators)  Principles and process for sharing power (transparency, decision making)  Participant involvement in research/methods (evaluation tools)  Gaps in research knowledge from CAB  Established history of partnership (time intensive) |
| A social network family-focused intervention to promote smoking cessation in Chinese and Vietnamese American male smokers: a feasibility study | Tsoh (2015) | - Social Cognitive Theory - Transtheoretical Model of Change - Social Network Theory | - Social-network family focused framework - Use of lay health workers (LHW) | - Design; Single-arm pilot (n=192) - Components: training of CHWs followed by 2 month family-focused smoking cessation program with follow up at 3 months | Intervention to promote smoking cessation among Chinese and Vietnamese smokers appears to be acceptable, feasible, and potentially efficacious. Significant improvements in SCT constructs knowledge, self-efficacy, and intention to quit. | - Lay health workers recruited smoker-family participants - Formative research was based on 39 in-depth dyadic and individual interviews with 13 smoker-family dyads (four Chinese and nine Vietnamese). - LHWs led outreach and provided supportive social network for smokers by involving the smoker’s family member and peers - Scripts for LHW in English, Chinese, and Vietnamese were used in concert with culturally appropriate graphics (no further detail) - Materials were field-tested in six focus groups (no further details) | Constructs informed activities and design  Constructs measured as outcomes  Use of Community Health Workers (informants, delivery)  Materials, activities, content reflect social norms and values (cultural relevance)  Multi-level intervention (individual, interpersonal)  Materials in 2+ languages |
| The iCook 4-H Study: an intervention and dissemination test of a youth/adult out-of-school program | White (2019) | - Social Cognitive Theory | - Community-Based Participatory Research | - Design: Randomized Controlled Trial in 5 states - Components: Six biweekly, two-hour sessions on cooking, eating, and physical activity & follow up communication. Evaluation at 0, 4, 12, and 24 months. | BMI increased compared with controls (p=.04). No significant differences in odds of being overweight or obese. Follow-up communication resulted in increased cooking skills and eating together (P=.08) among treatment families compared to controls. | - Each major study component (e.g., recruitment, curriculum development, measurement and evaluation) was managed by subcommittees (families, researchers, students, etc.) - CBPR used to gather feedback from stakeholders, session leaders, and target population (no further detail) - Community leaders were recruited to lead educational programs - Intervention development used cyclical and iterative processes throughout 6 years - SCT was operationalized by providing observational learning, reciprocal role modeling between adults and youths, and self-efficacy development and experiential learning | Use of Community Advisory Board (3)  Established history of partnership (trust, time intensive)  Use of Community Members, influential leaders (facilitators)  Constructs informed activities and design  Constructs measured as outcomes |
| Improving immunization rates through community-based participatory research: community health improvement for Milwaukee's children program | Willis (2016) | - Theory of Planned Behavior | - Community-Based Participatory Research - Knowledge-to-Action Framework | - Design: Cross-sectional pre-post-test design - Components: educational sessions, planned behavior change intervention, Social marketing campaign in target zip codes | Phase 1: Increases in immunization among the predominately black cohort were statistically significant.  Phase 2: Increases in immunization rates among the predominantly Hispanic cohort were not statistically significant. | - Pilot phase was 3 years and focused on community buy-in, awareness of the scope of the health disparities, and agreement regarding the research approach - Partners agreed upon an infrastructure to cultivate co-learning and power-sharing to address health disparities within specific zip codes - Phase two intervention was 5 years - CBPR approach was used to advance immunization health literacy in a central area of Milwaukee through the creation of co-learning opportunities. - 26-partner Community Advisory Board (CAB), included community members recruited from local CBOs, CBO representatives, and academic partners. - CAB worked closely with academic partners on the intervention’s structural and operational components - Workgroups were co-led by community members and academic partners who developed policies, outreach procedures, developed evaluation instruments and ensured cultural relevance of materials - Partner organizational improved their capacity building as a result of trainings with community members, which covered immunization plus areas of broader health disparity concerns, such as social determinants of health and advocating for system changes. - Trainings fostered bi-directional training, power-sharing, and learning opportunities between community and research members. - Four focus groups were conducted to assess barriers and facilitators which informed intervention activities - Accomodated for barriers participants faced (childcare, transportation, etc.) - Improved health literacy empowered families/caregivers and advanced a broader population impact that eclipsed its original goal – to increase immunizations. | Established history of partnership (time intensive)  Principles and process for sharing power (transparency, decision making, conflict resolution)  Use of Community Advisory Board (5)  Community-identified priorities  Multi-level intervention (individual, interpersonal, community, societal)  Capacity-building among community members  Co-learning mindset (co-leading, co-owning models)  Constructs informed activities and design  Materials, activities, content reflect social norms and values (cultural relevance)  Materials available in 2+ languages  Participant involvement in research/methods (evaluation tools)  Accommodations (transportation, childcare) |
